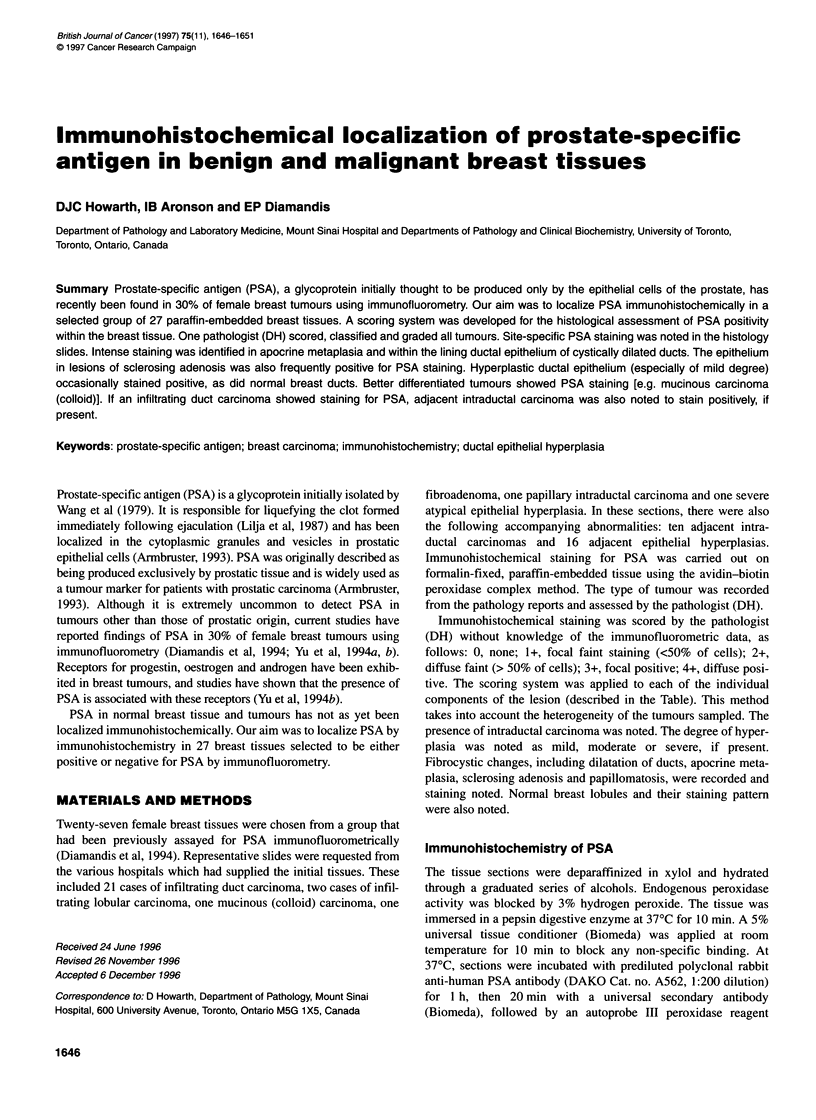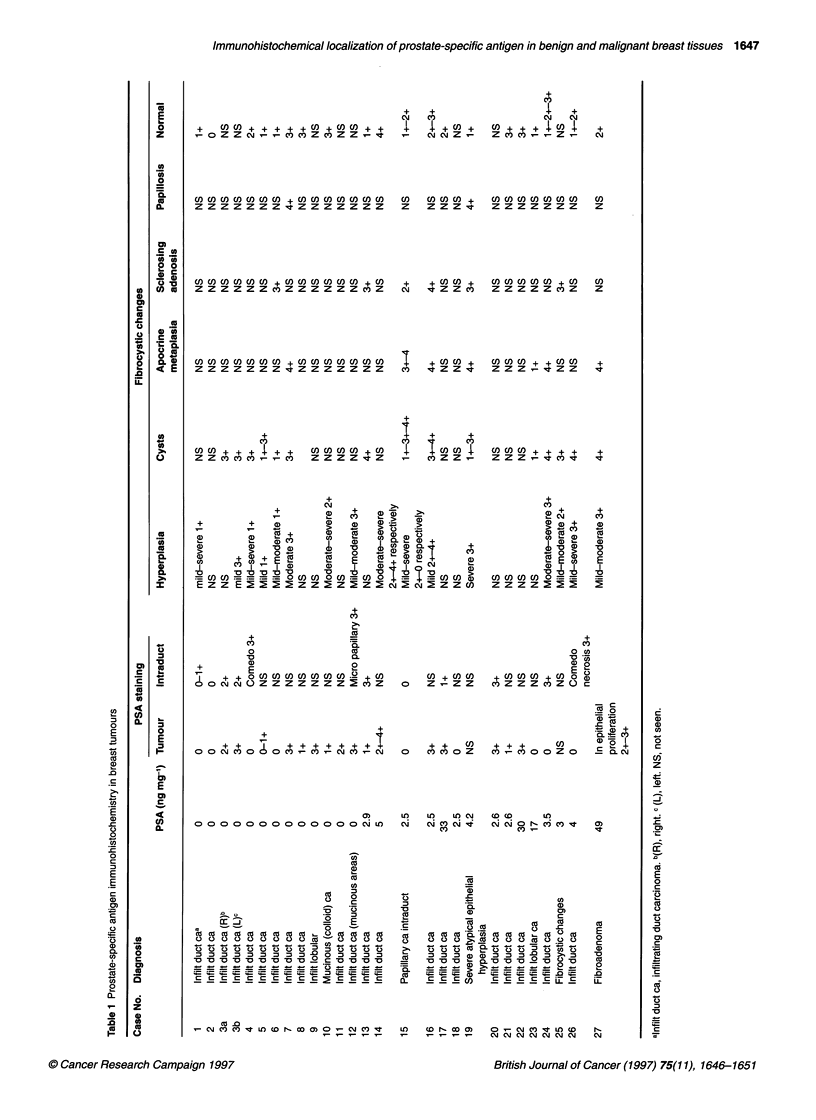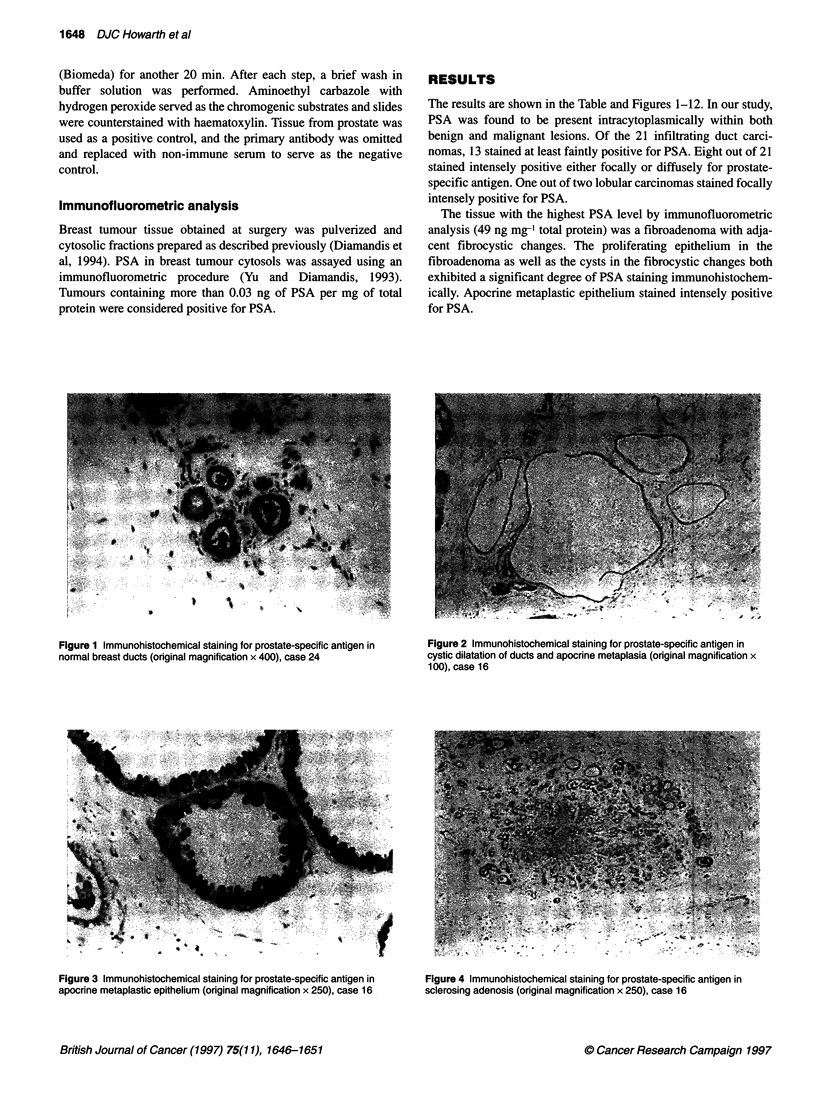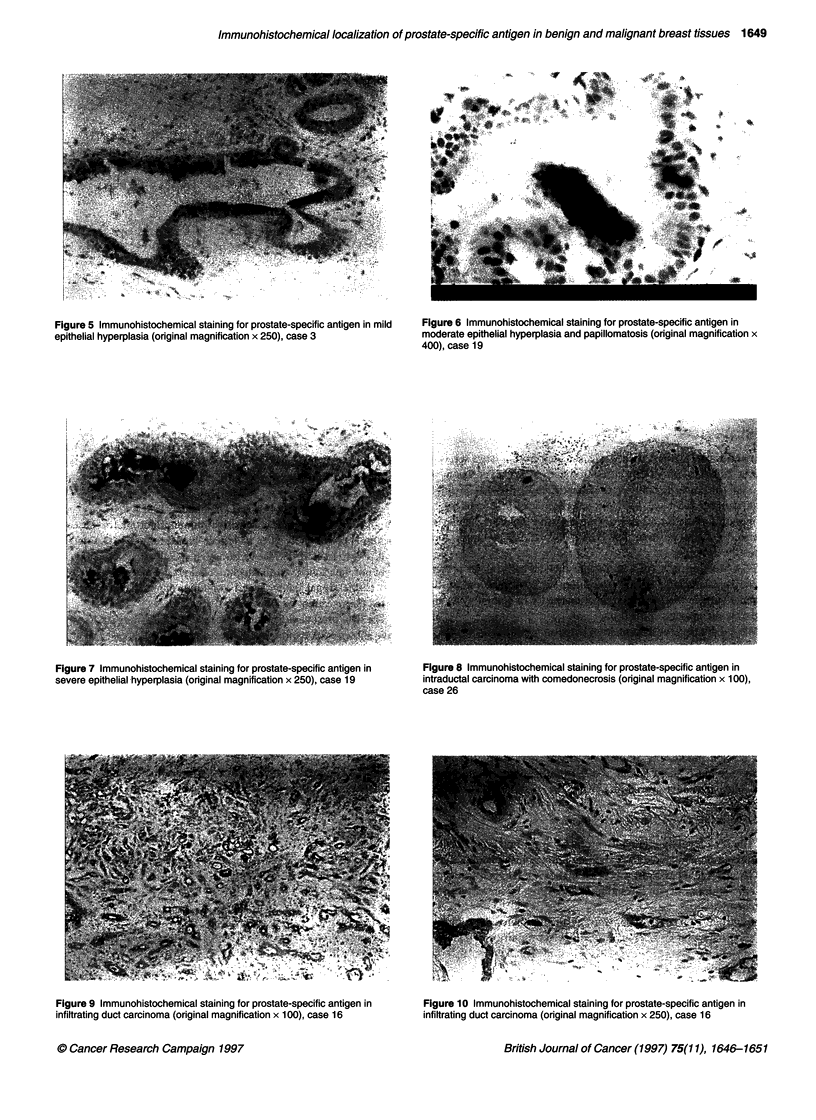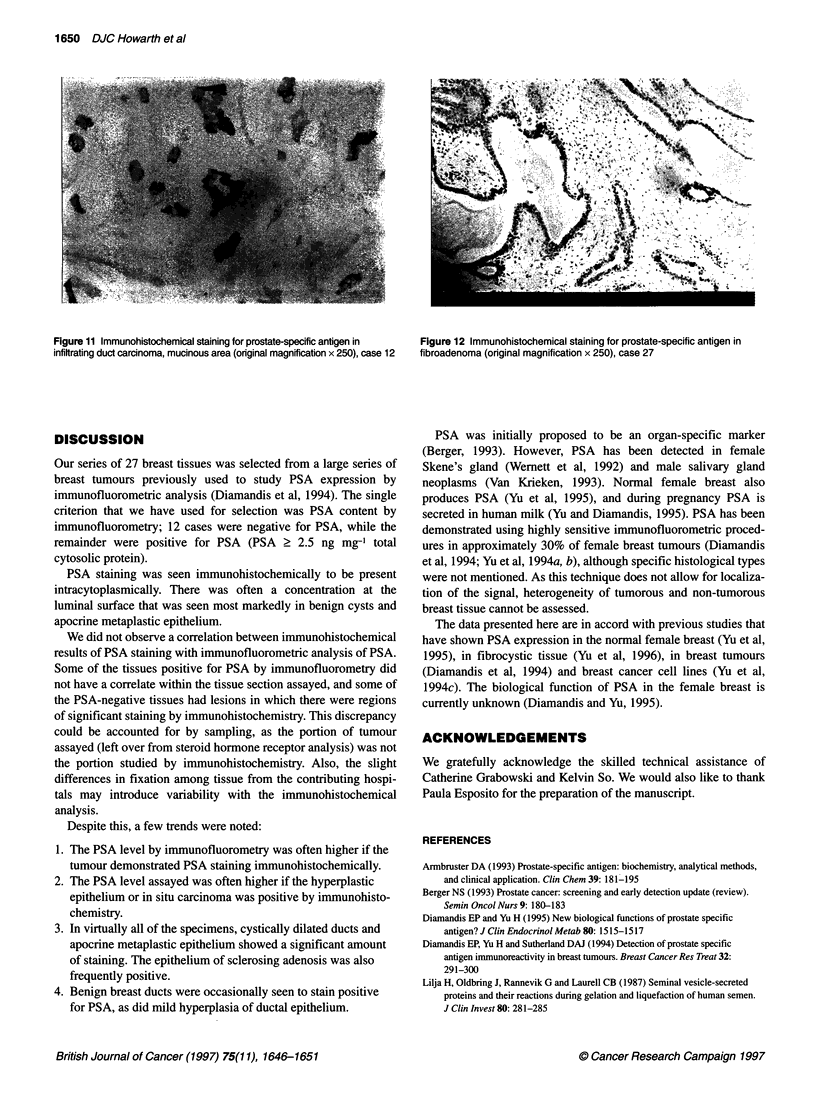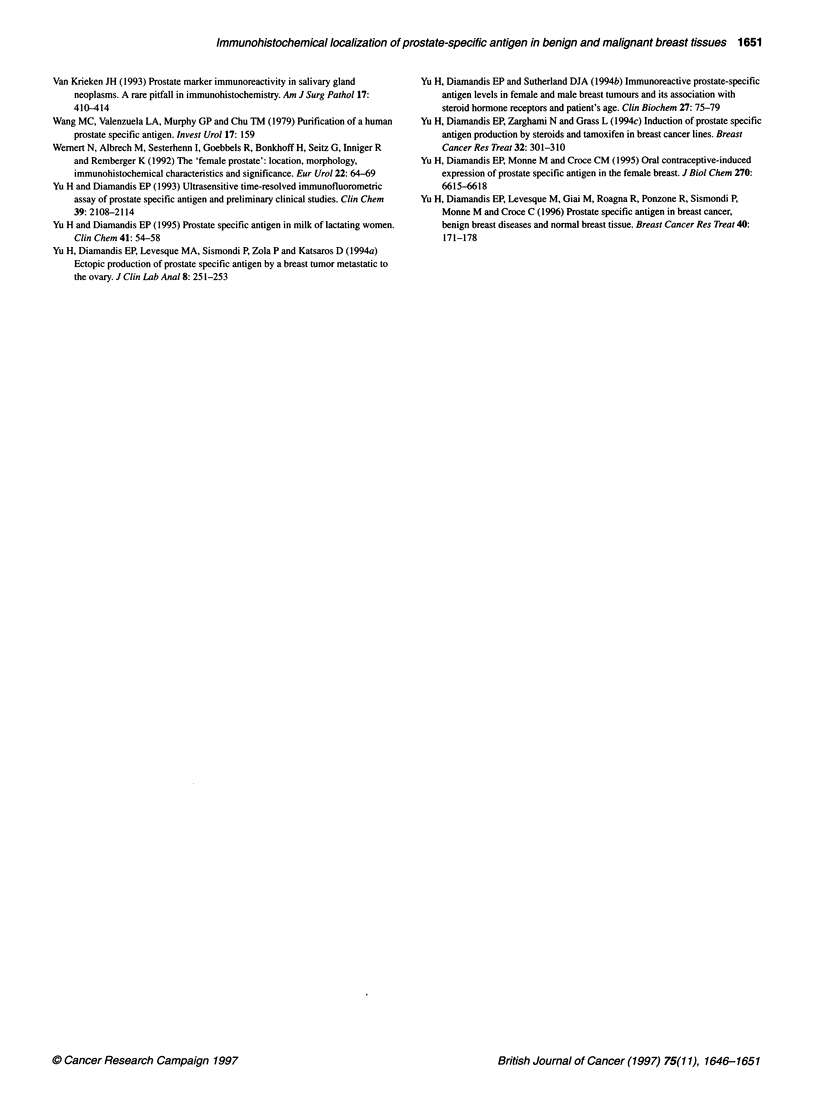# Immunohistochemical localization of prostate-specific antigen in benign and malignant breast tissues.

**DOI:** 10.1038/bjc.1997.280

**Published:** 1997

**Authors:** D. J. Howarth, I. B. Aronson, E. P. Diamandis

**Affiliations:** Department of Pathology and Laboratory Medicine, Mount Sinai Hospital, University of Toronto, Ontario, Canada.

## Abstract

**Images:**


British Journal of Cancer (1997) 75(11), 1646–1651
© 1997 Cancer Research Campaign

### Immunohistochemical localization of prostate-specific antigen in benign and malignant breast tissues

**DJC Howarth, IB Aronson and EP Diamandis**

Department of Pathology and Laboratory Medicine, Mount Sinai Hospital and Departments of Pathology and Clinical Biochemistry, University of Toronto, Toronto, Ontario, Canada

**Summary** Prostate-specific antigen (PSA), a glycoprotein initially thought to be produced only by the epithelial cells of the prostate, has recently been found in 30% of female breast tumours using immunofluorometry. Our aim was to localize PSA immunohistochemically in a selected group of 27 paraffin-embedded breast tissues. A scoring system was developed for the histological assessment of PSA positivity within the breast tissue. One pathologist (DH) scored, classified and graded all tumours. Site-specific PSA staining was noted in the histology slides. Intense staining was identified in apocrine metaplasia and within the lining ductal epithelium of cystically dilated ducts. The epithelium in lesions of sclerosing adenosis was also frequently positive for PSA staining. Hyperplastic ductal epithelium (especially of mild degree) occasionally stained positive, as did normal breast ducts. Better differentiated tumours showed PSA staining [e.g. mucinous carcinoma (colloid)]. If an infiltrating duct carcinoma showed staining for PSA, adjacent intraductal carcinoma was also noted to stain positively, if present.

**Keywords**: prostate-specific antigen; breast carcinoma; immunohistochemistry; ductal epithelial hyperplasia

Prostate-specific antigen (PSA) is a glycoprotein initially isolated by Wang et al (1979). It is responsible for liquefying the clot formed immediately following ejaculation (Lilja et al, 1987) and has been localized in the cytoplasmic granules and vesicles in prostatic epithelial cells (Armbruster, 1993). PSA was originally described as being produced exclusively by prostatic tissue and is widely used as a tumour marker for patients with prostatic carcinoma (Armbruster, 1993). Although it is extremely uncommon to detect PSA in tumours other than those of prostatic origin, current studies have reported findings of PSA in 30% of female breast tumours using immunofluorometry (Diamandis et al, 1994; Yu et al, 1994*a*, *b*). Receptors for progestin, oestrogen and androgen have been exhibited in breast tumours, and studies have shown that the presence of PSA is associated with these receptors (Yu et al, 1994*b*).

PSA in normal breast tissue and tumours has not as yet been localized immunohistochemically. Our aim was to localize PSA by immunohistochemistry in 27 breast tissues selected to be either positive or negative for PSA by immunofluorometry.

### MATERIALS AND METHODS

Twenty-seven female breast tissues were chosen from a group that had been previously assayed for PSA immunofluorometrically (Diamandis et al, 1994). Representative slides were requested from the various hospitals which had supplied the initial tissues. These included 21 cases of infiltrating duct carcinoma, two cases of infiltrating lobular carcinoma, one mucinous (colloid) carcinoma, one fibroadenoma, one papillary intraductal carcinoma and one severe atypical epithelial hyperplasia. In these sections, there were also the following accompanying abnormalities: ten adjacent intraductal carcinomas and 16 adjacent epithelial hyperplasias. Immunohistochemical staining for PSA was carried out on formalin-fixed, paraffin-embedded tissue using the avidin–biotin peroxidase complex method. The type of tumour was recorded from the pathology reports and assessed by the pathologist (DH).

Immunohistochemical staining was scored by the pathologist (DH) without knowledge of the immunofluorometric data, as follows: 0, none; 1+, focal faint staining (<50% of cells); 2+, diffuse faint (> 50% of cells); 3+, focal positive; 4+, diffuse positive. The scoring system was applied to each of the individual components of the lesion (described in the Table). This method takes into account the heterogeneity of the tumours sampled. The presence of intraductal carcinoma was noted. The degree of hyperplasia was noted as mild, moderate or severe, if present. Fibrocystic changes, including dilatation of ducts, apocrine metaplasia, sclerosing adenosis and papillomatosis, were recorded and staining noted. Normal breast lobules and their staining pattern were also noted.

### Immunohistochemistry of PSA

The tissue sections were deparaffinized in xylol and hydrated through a graduated series of alcohols. Endogenous peroxidase activity was blocked by 3% hydrogen peroxide. The tissue was immersed in a pepsin digestive enzyme at 37°C for 10 min. A 5% universal tissue conditioner (Biomeda) was applied at room temperature for 10 min to block any non-specific binding. At 37°C, sections were incubated with prediluted polyclonal rabbit anti-human PSA antibody (DAKO Cat. no. A562, 1:200 dilution) for 1 h, then 20 min with a universal secondary antibody (Biomeda), followed by an autoprobe III peroxidase reagent

*Received 24 June 1996*
*Revised 26 November 1996*
*Accepted 6 December 1996*

*Correspondence to:* D Howarth, Department of Pathology, Mount Sinai Hospital, 600 University Avenue, Toronto, Ontario M5G 1X5, Canada

**Table 1** Prostate-specific antigen immunohistochemistry in breast tumours

| Case No. | Diagnosis | | PSA staining | | Fibrocystic changes | | | | | |
|---|---|---|---|---|---|---|---|---|---|---|
| | | PSA (ng mg$^{-1}$) | Tumour | Intraduct | Hyperplasia | Cysts | Apocrine metaplasia | Sclerosing adenosis | Papillosis | Normal |
| 1 | Infilt duct ca[a] | 0 | 0 | 0–1+ | mild–severe 1+ | NS | NS | NS | NS | 1+ |
| 2 | Infilt duct ca | 0 | 0 | 0 | NS | NS | NS | NS | NS | 0 |
| 3a | Infilt duct ca (R)[b] | 0 | 2+ | 2+ | NS | 3+ | NS | NS | NS | NS |
| 3b | Infilt duct ca (L)[c] | 0 | 3+ | 2+ | mild 3+ | 3+ | NS | NS | NS | NS |
| 4 | Infilt duct ca | 0 | 0 | Comedo 3+ | Mild–severe 1+ | 3+ | NS | NS | NS | 2+ |
| 5 | Infilt duct ca | 0 | 0–1+ | NS | Mild 1+ | 1+–3+ | NS | NS | NS | 1+ |
| 6 | Infilt duct ca | 0 | 0 | NS | Mild–moderate 1+ | 1+ | NS | 3+ | NS | 1+ |
| 7 | Infilt duct ca | 0 | 3+ | NS | Moderate 3+ | 3+ | 4+ | NS | 4+ | 3+ |
| 8 | Infilt duct ca | 0 | 1+ | NS | NS | | NS | NS | NS | 3+ |
| 9 | Infilt lobular | 0 | 3+ | NS | NS | NS | NS | NS | NS | NS |
| 10 | Mucinous (colloid) ca | 0 | 1+ | NS | Moderate–severe 2+ | NS | NS | NS | NS | 3+ |
| 11 | Infilt duct ca | 0 | 2+ | NS | NS | NS | NS | NS | NS | NS |
| 12 | Infilt duct ca (mucinous areas) | 0 | 3+ | Micro papillary 3+ | Mild–moderate 3+ | NS | NS | NS | NS | NS |
| 13 | Infilt duct ca | 2.9 | 1+ | 3+ | NS | 4+ | NS | 3+ | NS | 1+ |
| 14 | Infilt duct ca | 5 | 2+–4+ | NS | Moderate–severe 2+–4+ respectively | NS | NS | NS | NS | 4+ |
| 15 | Papillary ca intraduct | 2.5 | 0 | 0 | Mild–severe 2+–0 respectively | 1+–3+–4+ | 3+–4 | 2+ | NS | 1+–2+ |
| 16 | Infilt duct ca | 2.5 | 3+ | NS | Mild 2+–4+ | 3+–4+ | 4+ | 4+ | NS | 2+–3+ |
| 17 | Infilt duct ca | 33 | 3+ | 1+ | NS | NS | NS | NS | NS | 2+ |
| 18 | Infilt duct ca | 2.5 | 0 | NS | NS | NS | NS | NS | NS | NS |
| 19 | Severe atypical epithelial hyperplasia | 4.2 | NS | NS | Severe 3+ | 1+–3+ | 4+ | 3+ | 4+ | 1+ |
| 20 | Infilt duct ca | 2.6 | 3+ | 3+ | NS | NS | NS | NS | NS | NS |
| 21 | Infilt duct ca | 2.6 | 1+ | NS | NS | NS | NS | NS | NS | 3+ |
| 22 | Infilt duct ca | 30 | 3+ | NS | NS | NS | NS | NS | NS | 3+ |
| 23 | Infilt lobular ca | 17 | 0 | NS | NS | 1+ | 1+ | NS | NS | 1+ |
| 24 | Infilt duct ca | 3.5 | 0 | 3+ | Moderate–severe 3+ | 4+ | 4+ | NS | NS | 1+–2+–3+ |
| 25 | Fibrocystic changes | 3 | NS | NS | Mild–moderate 2+ | 3+ | NS | 3+ | NS | NS |
| 26 | Infilt duct ca | 4 | 0 | Comedo necrosis 3+ | Mild–severe 3+ | 4+ | NS | NS | NS | 1+–2+ |
| 27 | Fibroadenoma | 49 | In epithelial proliferation 2+–3+ | | Mild–moderate 3+ | 4+ | 4+ | NS | NS | 2+ |

[a]Infilt duct ca, infiltrating duct carcinoma. [b](R), right. [c] (L), left. NS, not seen.

© Cancer Research Campaign 1997

(Biomeda) for another 20 min. After each step, a brief wash in buffer solution was performed. Aminoethyl carbazole with hydrogen peroxide served as the chromogenic substrates and slides were counterstained with haematoxylin. Tissue from prostate was used as a positive control, and the primary antibody was omitted and replaced with non-immune serum to serve as the negative control.

### Immunofluorometric analysis

Breast tumour tissue obtained at surgery was pulverized and cytosolic fractions prepared as described previously (Diamandis et al, 1994). PSA in breast tumour cytosols was assayed using an immunofluorometric procedure (Yu and Diamandis, 1993). Tumours containing more than 0.03 ng of PSA per mg of total protein were considered positive for PSA.

### RESULTS

The results are shown in the Table and Figures 1–12. In our study, PSA was found to be present intracytoplasmically within both benign and malignant lesions. Of the 21 infiltrating duct carcinomas, 13 stained at least faintly positive for PSA. Eight out of 21 stained intensely positive either focally or diffusely for prostate-specific antigen. One out of two lobular carcinomas stained focally intensely positive for PSA.

The tissue with the highest PSA level by immunofluorometric analysis (49 ng $mg^{-1}$ total protein) was a fibroadenoma with adjacent fibrocystic changes. The proliferating epithelium in the fibroadenoma as well as the cysts in the fibrocystic changes both exhibited a significant degree of PSA staining immunohistochemically. Apocrine metaplastic epithelium stained intensely positive for PSA.

**Figure 1** Immunohistochemical staining for prostate-specific antigen in normal breast ducts (original magnification × 400), case 24

**Figure 2** Immunohistochemical staining for prostate-specific antigen in cystic dilatation of ducts and apocrine metaplasia (original magnification × 100), case 16

**Figure 3** Immunohistochemical staining for prostate-specific antigen in apocrine metaplastic epithelium (original magnification × 250), case 16

**Figure 4** Immunohistochemical staining for prostate-specific antigen in sclerosing adenosis (original magnification × 250), case 16

© Cancer Research Campaign 1997

**Figure 5** Immunohistochemical staining for prostate-specific antigen in mild epithelial hyperplasia (original magnification × 250), case 3

**Figure 6** Immunohistochemical staining for prostate-specific antigen in moderate epithelial hyperplasia and papillomatosis (original magnification × 400), case 19

**Figure 7** Immunohistochemical staining for prostate-specific antigen in severe epithelial hyperplasia (original magnification × 250), case 19

**Figure 8** Immunohistochemical staining for prostate-specific antigen in intraductal carcinoma with comedonecrosis (original magnification × 100), case 26

**Figure 9** Immunohistochemical staining for prostate-specific antigen in infiltrating duct carcinoma (original magnification × 100), case 16

**Figure 10** Immunohistochemical staining for prostate-specific antigen in infiltrating duct carcinoma (original magnification × 250), case 16

**Figure 11** Immunohistochemical staining for prostate-specific antigen in infiltrating duct carcinoma, mucinous area (original magnification × 250), case 12

**Figure 12** Immunohistochemical staining for prostate-specific antigen in fibroadenoma (original magnification × 250), case 27

### DISCUSSION

Our series of 27 breast tissues was selected from a large series of breast tumours previously used to study PSA expression by immunofluorometric analysis (Diamandis et al, 1994). The single criterion that we have used for selection was PSA content by immunofluorometry; 12 cases were negative for PSA, while the remainder were positive for PSA (PSA $\geq$ 2.5 ng mg$^{-1}$ total cytosolic protein).

PSA staining was seen immunohistochemically to be present intracytoplasmically. There was often a concentration at the luminal surface that was seen most markedly in benign cysts and apocrine metaplastic epithelium.

We did not observe a correlation between immunohistochemical results of PSA staining with immunofluorometric analysis of PSA. Some of the tissues positive for PSA by immunofluorometry did not have a correlate within the tissue section assayed, and some of the PSA-negative tissues had lesions in which there were regions of significant staining by immunohistochemistry. This discrepancy could be accounted for by sampling, as the portion of tumour assayed (left over from steroid hormone receptor analysis) was not the portion studied by immunohistochemistry. Also, the slight differences in fixation among tissue from the contributing hospitals may introduce variability with the immunohistochemical analysis.

Despite this, a few trends were noted:

1. The PSA level by immunofluorometry was often higher if the tumour demonstrated PSA staining immunohistochemically.
2. The PSA level assayed was often higher if the hyperplastic epithelium or in situ carcinoma was positive by immunohistochemistry.
3. In virtually all of the specimens, cystically dilated ducts and apocrine metaplastic epithelium showed a significant amount of staining. The epithelium of sclerosing adenosis was also frequently positive.
4. Benign breast ducts were occasionally seen to stain positive for PSA, as did mild hyperplasia of ductal epithelium.

PSA was initially proposed to be an organ-specific marker (Berger, 1993). However, PSA has been detected in female Skene's gland (Wernett et al, 1992) and male salivary gland neoplasms (Van Krieken, 1993). Normal female breast also produces PSA (Yu et al, 1995), and during pregnancy PSA is secreted in human milk (Yu and Diamandis, 1995). PSA has been demonstrated using highly sensitive immunofluorometric procedures in approximately 30% of female breast tumours (Diamandis et al, 1994; Yu et al, 1994*a*, *b*), although specific histological types were not mentioned. As this technique does not allow for localization of the signal, heterogeneity of tumorous and non-tumorous breast tissue cannot be assessed.

The data presented here are in accord with previous studies that have shown PSA expression in the normal female breast (Yu et al, 1995), in fibrocystic tissue (Yu et al, 1996), in breast tumours (Diamandis et al, 1994) and breast cancer cell lines (Yu et al, 1994*c*). The biological function of PSA in the female breast is currently unknown (Diamandis and Yu, 1995).

### ACKNOWLEDGEMENTS

We gratefully acknowledge the skilled technical assistance of Catherine Grabowski and Kelvin So. We would also like to thank Paula Esposito for the preparation of the manuscript.

### REFERENCES

Armbruster DA (1993) Prostate-specific antigen: biochemistry, analytical methods, and clinical application. *Clin Chem* **39**: 181–195

Berger NS (1993) Prostate cancer: screening and early detection update (review). *Semin Oncol Nurs* **9**: 180–183

Diamandis EP and Yu H (1995) New biological functions of prostate specific antigen? *J Clin Endocrinol Metab* **80**: 1515–1517

Diamandis EP, Yu H and Sutherland DAJ (1994) Detection of prostate specific antigen immunoreactivity in breast tumours. *Breast Cancer Res Treat* **32**: 291–300

Lilja H, Oldbring J, Rannevik G and Laurell CB (1987) Seminal vesicle-secreted proteins and their reactions during gelation and liquefaction of human semen. *J Clin Invest* **80**: 281–285

© Cancer Research Campaign 1997

Van Krieken JH (1993) Prostate marker immunoreactivity in salivary gland neoplasms. A rare pitfall in immunohistochemistry. *Am J Surg Pathol* **17**: 410–414

Wang MC, Valenzuela LA, Murphy GP and Chu TM (1979) Purification of a human prostate specific antigen. *Invest Urol* **17**: 159

Wernert N, Albrech M, Sesterhenn I, Goebbels R, Bonkhoff H, Seitz G, Inniger R and Remberger K (1992) The 'female prostate': location, morphology, immunohistochemical characteristics and significance. *Eur Urol* **22**: 64–69

Yu H and Diamandis EP (1993) Ultrasensitive time-resolved immunofluorometric assay of prostate specific antigen and preliminary clinical studies. *Clin Chem* **39**: 2108–2114

Yu H and Diamandis EP (1995) Prostate specific antigen in milk of lactating women. *Clin Chem* **41**: 54–58

Yu H, Diamandis EP, Levesque MA, Sismondi P, Zola P and Katsaros D (1994*a*) Ectopic production of prostate specific antigen by a breast tumor metastatic to the ovary. *J Clin Lab Anal* **8**: 251–253

Yu H, Diamandis EP and Sutherland DJA (1994*b*) Immunoreactive prostate-specific antigen levels in female and male breast tumours and its association with steroid hormone receptors and patient's age. *Clin Biochem* **27**: 75–79

Yu H, Diamandis EP, Zarghami N and Grass L (1994*c*) Induction of prostate specific antigen production by steroids and tamoxifen in breast cancer lines. *Breast Cancer Res Treat* **32**: 301–310

Yu H, Diamandis EP, Monne M and Croce CM (1995) Oral contraceptive-induced expression of prostate specific antigen in the female breast. *J Biol Chem* **270**: 6615–6618

Yu H, Diamandis EP, Levesque M, Giai M, Roagna R, Ponzone R, Sismondi P, Monne M and Croce C (1996) Prostate specific antigen in breast cancer, benign breast diseases and normal breast tissue. *Breast Cancer Res Treat* **40**: 171–178

© Cancer Research Campaign 1997